# Application of norepinephrine in the treatment of septic shock: a meta-analysis

**DOI:** 10.1007/s11845-024-03827-x

**Published:** 2024-11-09

**Authors:** Qiu Ying Xu, Yan Hong Jin, Li Fu, Ying Ying Li

**Affiliations:** Department of Intensive Care Unit, Beijing Friendship Hospital, Capital Medical University, No. 95 Yong’an Road, Xicheng District, Beijing, 100050 China

**Keywords:** Meta-analysis, Norepinephrine, Randomized controlled trial, Septic shock

## Abstract

**Objective:**

To systematically evaluate the efficacy and safety of norepinephrine in the treatment of septic shock.

**Methods:**

Literature retrieval of eligible randomized controlled trials (RCTs) on norepinephrine in the treatment of septic shock was performed in three English databases including PubMed, Web of Science, and Medline from database establishment to October 1, 2023. The Cochrane risk bias tool was used to evaluate the quality of the included literature. RevMan 5.3 software was used for meta-analysis.

**Results:**

A total of 14 RCTs were included in this study, and the risk of bias was low. Our meta-analysis showed that the norepinephrine group had significantly better outcomes in reducing the 28-day mortality rate (RR = 0.92; 95% CI, 0.86 ~ 0.99; *P* = 0.03), the incidence of arrhythmia (RR = 0.54; 95% CI, 0.45 ~ 0.64; *P* < 0.0001), and the length of stay in intensive care unit (ICU) (MD =  − 1.03; 95% CI, − 1.85 to approximately − 0.21; *P* = 0.01) than those of the control group. However, there were no statistically significant differences in in-hospital mortality rate (RR = 0.97; 95% CI, 0.90 ~ 1.04; *P* = 0.4), the 90-day mortality rate (RR = 1.07; 95% CI, 0.97 ~ 1.18; *P* = 0.15), length of hospital stay (MD = 0.03; 95% CI, − 1.13 ~ 1.18; *P* = 0.96), and the rate of achieving target MAP (RR = 1.27; 95% CI, 0.72 ~ 2.26; *P* = 0.41) between the norepinephrine group and the control group.

**Conclusion:**

Norepinephrine has the advantages of improving 28-day mortality, shortening ICU hospitalization time, and reducing the incidence of arrhythmia. It is a more effective choice for the treatment of septic shock than other vasopressors, and the incidence of arrhythmia is low.

## Introduction

Sepsis remains a serious cause of morbidity and mortality in critically ill patients worldwide, despite the use of broad-spectrum antibiotics, advanced intensive care unit (ICU) management, as well as resuscitation strategies and protocols [1]. The global burden of sepsis is estimated at > 19 million cases each year and 5 million sepsis-related deaths annually, especially in low-income and middle-income countries [2]. Septic shock, the most severe complication of sepsis, accounts for 10% of all admissions to the ICU, with a related mortality rate of 40–60% [3]. Septic shock is now defined as a life-threatening organ dysfunction caused by a dysregulated host response to infection [4]. It is mainly manifested as severe hypotension with low systolic blood pressure ≤ 90 mmHg (or mean arterial blood pressure ≤ 65 mmHg), accompanied by hypoperfusion [5].

The current practice in the treatment of sepsis is to improve tissue oxygenation and perfusion in patients, along with appropriate administration of antibiotics against pathogenic microorganisms. According to Guidelines 2021 for sepsis [6], for adult septic shock patients, it is recommended to prioritize norepinephrine as a vasopressor rather than other vasopressor agents, and adrenaline or dopamine can be used as an alternative option in cases where norepinephrine cannot be obtained. Meanwhile, vasopressors should be immediately initiated in severe hypotension with life-threatening septic shock [7, 8]. For septic shock, vasoactive agents are an important means of maintaining hemodynamic stability and ensuring perfusion of major organs [7].

A previous meta-analysis in 2018 analyzed outcomes such as oxygen transport, oxygen consumption, cardiac index, cardiac ratio, MAP, mean pulmonary artery pressure, central venous pressure, and systemic vascular resistance index to clarify the role of norepinephrine and other vasoactive agents in maintaining hemodynamic stability and ensuring perfusion of major organs [9]. In order to further investigate the differences in efficacy and safety between norepinephrine and other vasoactive agents or placebo in patients with septic shock, the present study included more updated trials for meta-analysis on the patients’ outcomes, such as mortality rate, length of hospital stay, and incidence of adverse events. Add more references for norepinephrine and other vasoactive drugs or placebos in the treatment of patients with septic shock in efficacy and safety.

## Materials and methods

### Retrieval strategies

A systematic literature retrieval was performed in three English databases including PubMed, Web of Science, and Medline from database establishment to October 1, 2023, using the keywords “semantic shock” OR “sepsis” OR “multiple sepsis” OR “imperfect shock” AND “norepinephrine” OR “noradrenaline.” Besides focusing on the retrieval of titles, more literature was searched based on the references of relevant literature retrieved.

### Inclusion and exclusion criteria

Inclusion criteria are as follows: (1) research type of RCTs; (2) research subjects of patients with septic shock; (3) intervention using norepinephrine, with the use of placebo or other vasopressor agents as the control; and (4) outcome measures of 28-day mortality rate, in-hospital mortality rate, 90-day mortality rate, length of hospital stay, length of stay in ICU, the incidence of arrhythmia, and the rate of achieving target MAP.

Exclusion criteria are as follows: (1) non-population-based study; (2) conference abstracts, case reports, systematic reviews, etc.; (3) insufficient outcome data that cannot be analyzed; (4) duplicates; (5) incomplete studies; (6) non-randomized controlled trials.

### Literature screening and data extraction

Literature screening was performed independently by two researchers based on inclusion and exclusion criteria. After preliminary screening by reading titles and abstracts, studies that might meet the inclusion criteria were checked through full-text reading. Any disagreement between the two researchers was resolved by discussion until a consensus was reached or by consulting a third researcher. After literature screening, two researchers would extract data (literature information, research type, research time, demographic characteristics of subjects, sample size of two groups, control type, and primary outcome measures) according to the established standard data extraction table.

### Quality evaluation

The risk of bias assessment of the included studies was assessed using the bias risk assessment tool for RCTs recommended by the Cochrane Guidelines [10]. Evaluation of the risk of bias covered seven items including random sequence generation, allocation concealment, blinding of participants, personnel, and outcome assessors, completeness of outcome data, selective reporting of results, and others. The evaluation results of each item were divided into three levels: low risk, unclear, and high risk, and finally represented by a risk of bias graph. Two researchers independently evaluated the risk of bias in the included studies using standardized data extraction tables. Any discrepancies were solved by discussion with a third researcher until consensus was achieved.

### Statistical analysis

RevMan 5.3 software was used for statistical analysis. For outcome variables (length of hospital stay and length of stay in ICU) calculated using the same criteria among continuous variables, mean difference (MD) was used as the statistical measure, while for the binary variable (28-day mortality rate, in-hospital mortality rate, 90-day mortality rate, incidence of arrhythmia, or the rate of achieving target MAP), the risk ratio (RR) was the statistical measure. The 95% confidence interval (CI) was provided for each statistic. The heterogeneity test was realized by using ${I}^{2}$ statistics to quantify the heterogeneity. If the hypothesis of homogeneity was not rejected (${I}^{2}$ < 50% or *P* > 0.1), a fixed-effect model was used for calculation; otherwise, a random-effect model was used due to poor homogeneity (${I}^{2}$ > 50% or *P* ≤ 0.1). The inspection level of the meta-analysis was set at 0.05.

## Results

### Literature screening process and results

**Fig. 1:**
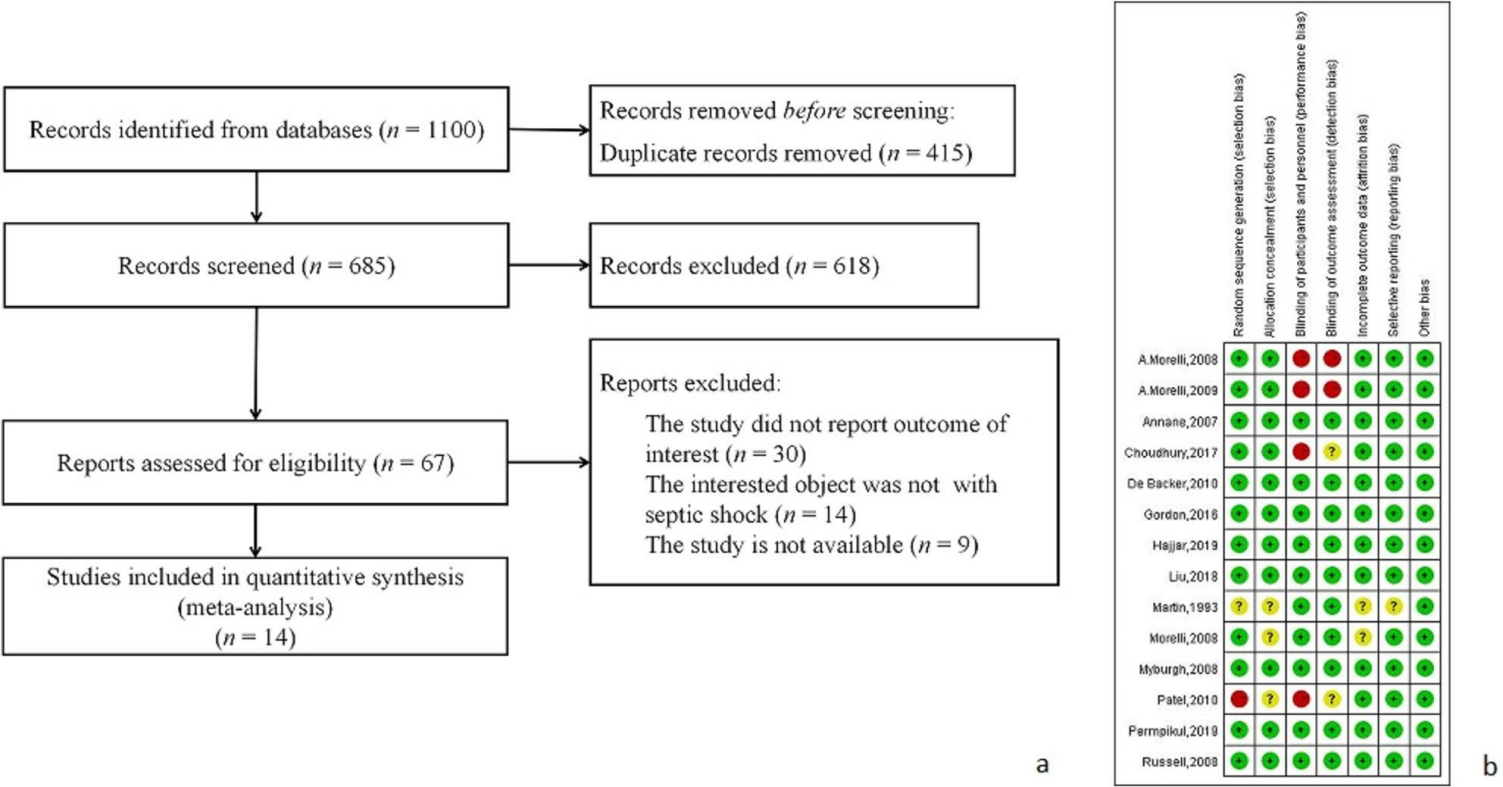
**a** The flow chart of included studies. **b** Risk of bias summary

A total of 685 articles were obtained in initial retrieval, and finally, 14 RCTs were included for meta-analysis after subsequent screenings. The process and results of literature retrieval are shown in Fig. 1a.

### Basic characteristics of the included studies and assessment of the risk of bias

All 14 eligible RCTs, involving a total of 5028 cases, were included for meta-analysis. Most of the enrolled literature were multicenter studies, primarily conducted in the ICU, with one study involving patients from the emergency department of the hospital. The major subjects included in the study were the population aged 40–70 years old. In each study, both the norepinephrine group and the control group included male and female cases, without statistically significant difference in the gender ratio between groups, while a small number of studies did not report the gender ratio of the two groups separately. The controls included in the study used placebo, terlipressin, adrenaline, dopamine, phenylephrine, etc. In addition, the included studies had a lower risk of bias, with the inclusion of subjects randomly during experimental design and evaluation of the efficacy and safety of norepinephrine objectively. Detailed basic characteristics of all the included studies are shown in Table 1 [11–24], and the assessment of the risk of bias is shown in Fig. 1b.

**Table 1:** Characteristics of included studies

No	Country	Department	Age of intervention group and control group	Sample size of intervention group and control group (male/female)	Control intervention	Primary outcome
A. Morelli, 2008 [11]	Italy	ICU	67/66	14/6 13/6	Terlipressin	Hemodynamic changes
A. Morelli, 2009 [12]	Italy	ICU	64/67	12/3 10/5	Terlipressin	Hemodynamic changes
Annane, 2007 [13]	France	Multicenter study involving 19 ICUs	60/65	99/70 103/58	Adrenaline ± placebo	28-day all-cause mortality rate
Choudhury, 2017 [14]	India	ICU	48 ± 29.12/46 ± 76.12	34/8 35/7	Terlipressin	Qualified hemodynamics
De Backer, 2010 [15]	Belgium, Austria, and Spain	Multicenter study involving 8 ICUs	67/68	449/372 507/351	Dopamine	28-day mortality rate
Gordon, 2016 [16]	Britain	Multicenter study involving 18 ICUs	66	238/171	Antidiuretic hormone	Days without renal failure within 28 days
Hajjar, 2019 [17]	Brazil	ICU	62/64	69/56 68/57	Vasopressin	28-day mortality rate
Liu, 2018 [18]	China	Multicenter study involving 21 ICUs	61.09/16.20; 60.93/15.86	169/97 162/98	Terlipressin	28-day all-cause mortality rate
Martin, 1993 [19]	France	ICU	52 ± 12/53 ± 19	12/4 12/4	Dopamine	Qualified hemodynamics
Morelli, 2008 [20]	Italy	ICU	70/70	9/7 12/4	Phenylephedrine	Qualified hemodynamics
Myburgh, 2008 [21]	Australia	Multicenter study involving 4 ICUs	60.4 ± 14.8/59.4 ± 15.9	82/56 85/54	Adrenaline	Qualified hemodynamics
Patel, 2010 [22]	The USA	ICU	Unknown	52/66 64/70	Dopamine	28-day all-cause mortality rate
Permpikul, 2019 [23]	Thailand	Emergency department	65/68	71/84 77/78	Placebo	Shock control rate within 6 h
Russell, 2008 [24]	Canada, Australia, and the USA	Multicenter study involving 27 hospitals	61.8 ± 16/59.3 ± 16.4	229/153 246/150	Vasopressin	All-cause mortality rate

The risk of included literature was assessed according to the Cochrane bias risk assessment tool. Four articles were rated as high risk of bias, two as moderate risk of bias, and eight as low risk of bias. The literature evaluation basis is as follows: (1) In terms of random sequence generation, 12 reports used the random sequence generation method to be rated as low risk; one article did not mention that the random method was evaluated as the risk of bias was not clear; one article was not randomly rated as high-risk bias. (2) In terms of allocation concealment, 12 articles implemented allocation concealment and were rated as low risk of bias; two articles did not report allocation concealment, and the risk of bias was unclear. (3) In terms of the blind method of subjects and researchers, four studies did not use the blind method for subjects and researchers and were rated as high risk of bias; ten studies were double-blinded and rated as low-risk bias. (4) In terms of blinding of outcome evaluators, two studies did not use blinding for outcome evaluators, which was rated as high risk of bias; two studies were not mentioned, and the risk of bias was not clear. Ten studies also used blinding for outcome evaluators, which was rated as low risk of bias. (5) In terms of incomplete data results, 12 articles showed complete data and were rated as low-risk bias; two studies did not mention issues such as loss of follow-up, and the risk of bias was unclear. (6) In terms of selective reporting of results, 13 studies did not find selective reporting of results, which was rated as low risk of bias; one study did not clarify the pre-study indicators, the selective reporting of the results was unclear, and the risk of bias was unclear. (7) In terms of other sources of bias, 14 studies found no other bias and were rated as low risk of bias.

### Meta-analysis

#### Effect of norepinephrine on 28-day mortality rate in patients

**Fig. 2:**
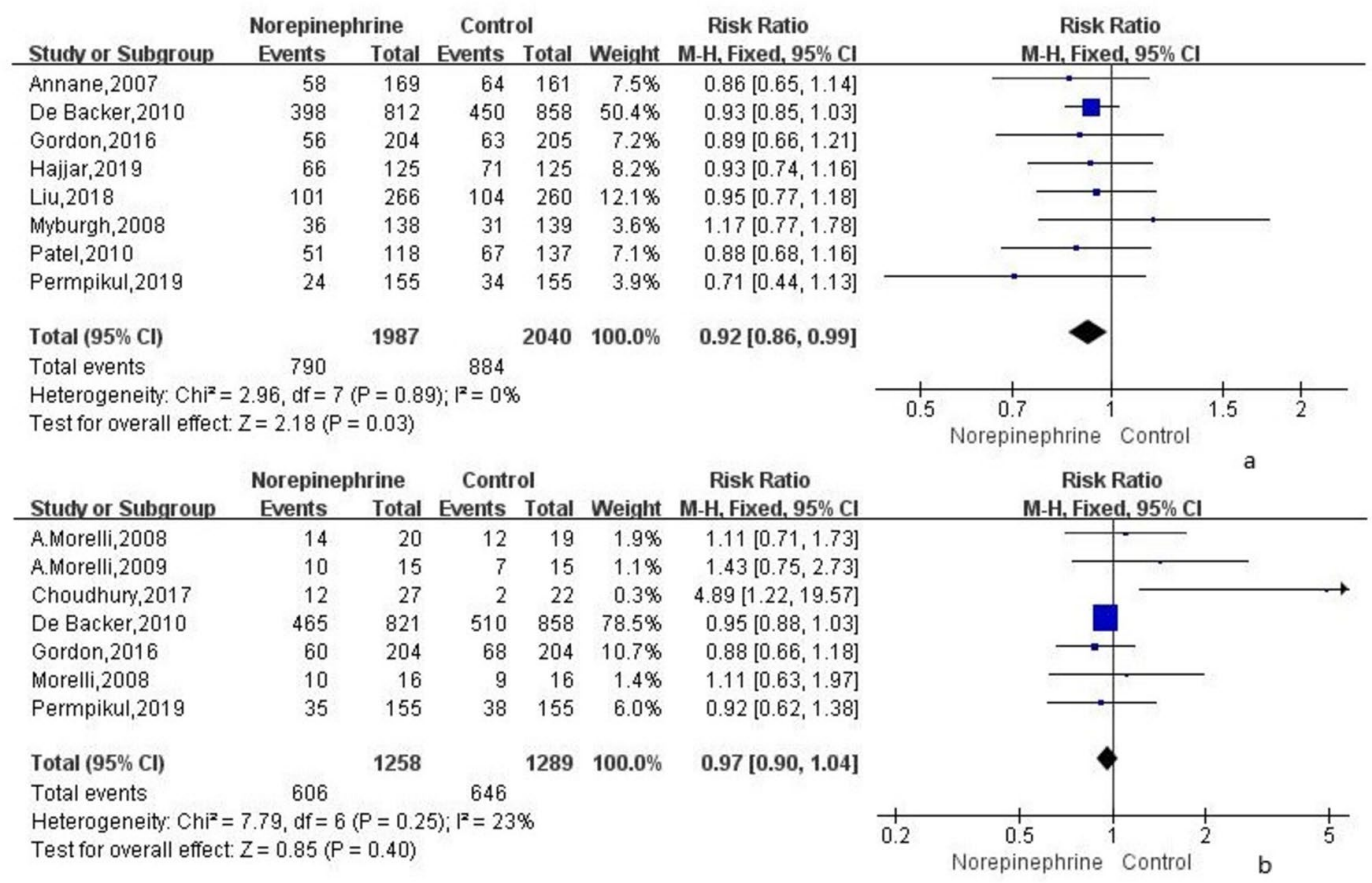
**a** The effect of norepinephrine on 28-day mortality. **b** The effect of norepinephrine on hospital mortality

The 28-day mortality rate was analyzed in 8 RCTs. The overall analysis using a fixed-effect model (${I}^{2}$ = 0%) showed that the 28-day mortality rate in the norepinephrine group was significantly lower than that in the control group (RR = 0.92; 95% CI, 0.86 ~ 0.99; *P* = 0.03; Fig. 2a).

#### Effect of norepinephrine on in-hospital mortality rate in patients

The in-hospital mortality rate in 7 RCTs using the fixed-effect model (${I}^{2}$ = 23%) revealed that it was lower in the norepinephrine group than in the control group, yet without statistical significance (RR = 0.97; 95% CI, 0.90 ~ 1.04; *P* = 0.4; Fig. 2b).

#### Effect of norepinephrine on 90-day mortality rate in patients

**Fig. 3:**
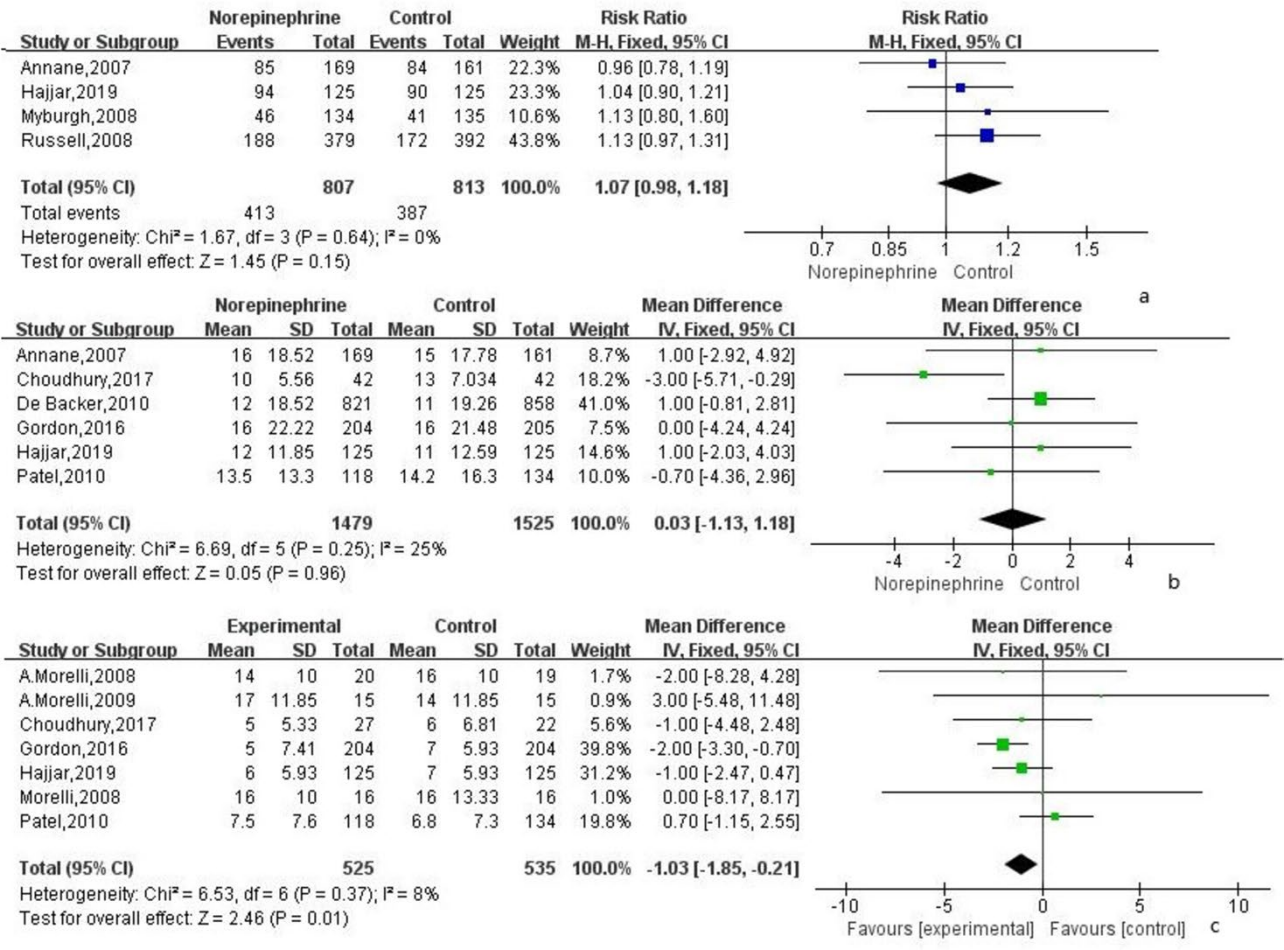
**a** The effect of norepinephrine on 90-day mortality. **b** The effect of norepinephrine on hospital length of stay. **c** The effect of norepinephrine on ICU length of stay

The 90-day mortality rate analyzed in 4 RCTs using the fixed-effect model (${I}^{2}$ = 0%) indicated a lower rate in the norepinephrine group than that in the control group, without statistical significance (RR = 1.07; 95% CI, 0.98 ~ 1.18; *P* = 0.15; Fig. 3a).

#### Effect of norepinephrine on length of hospital stay in patients

The length of hospital stay was analyzed in 6 RCTs. The overall analysis using the fixed-effect model (${I}^{2}$ = 25%) showed no difference in the length of hospital stay between the norepinephrine group and the control group (MD = 0.03; 95% CI, − 1.13 ~ 1.18; *P* = 0.96; Fig. 3b).

#### Effect of norepinephrine on length of stay in ICU in patients

The analysis was also performed in 7 RCTs by employing a fixed-effect model (${I}^{2}$ = 8%). It was found that the length of stay in the ICU of the norepinephrine group was significantly less than that of the control group (MD =  − 1.03; 95% CI, − 1.85 to approximately − 0.21; *P* = 0.01; Fig. 3c).

#### Effect of norepinephrine on the incidence of arrhythmia in patients

**Fig. 4:**
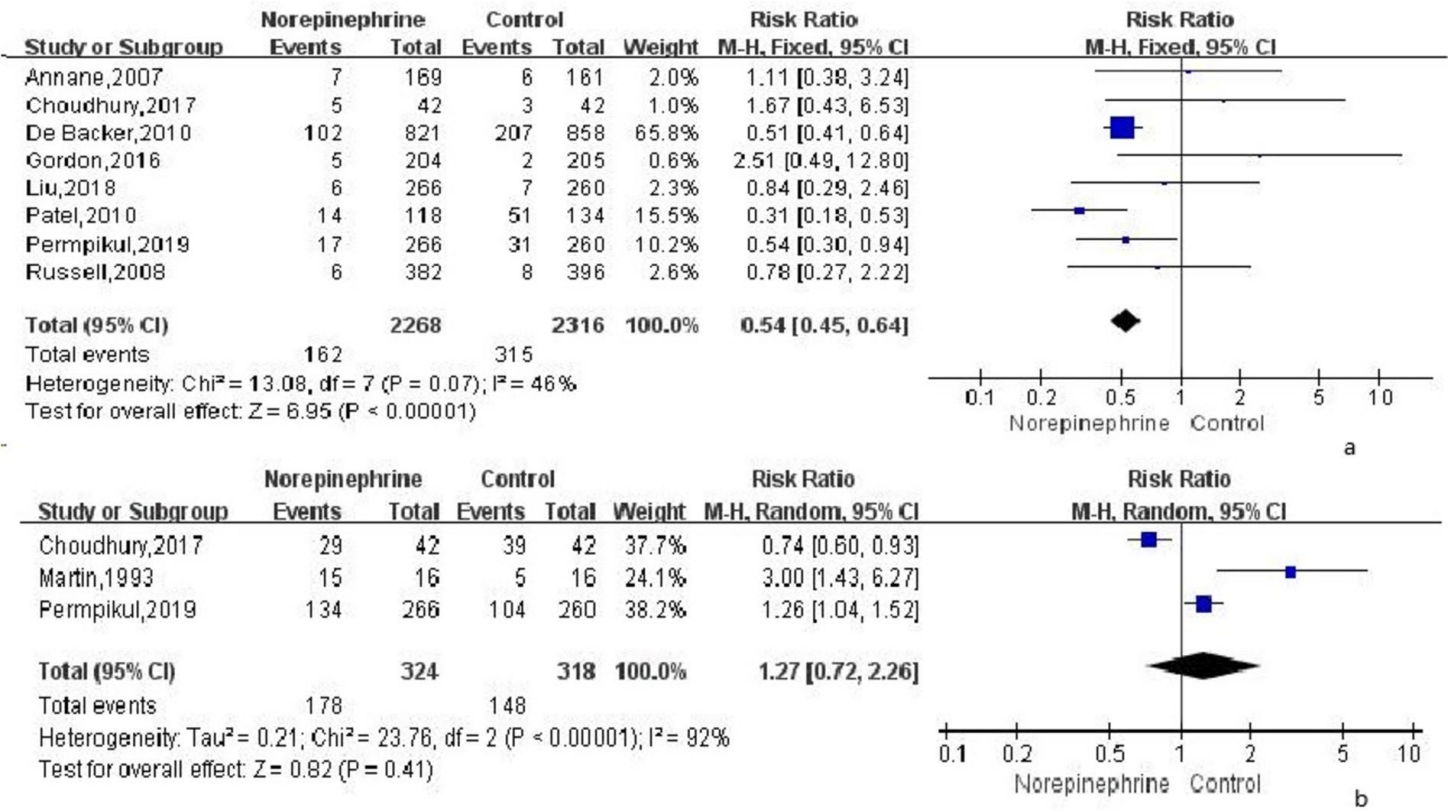
**a** The effect of norepinephrine on the incidence of arrhythmia. **b** The effect of norepinephrine on the rate of achieved the target mean arterial pressure

By using a fixed-effect model (${I}^{2}$ = 46%) for analysis in 8 RCTs, the incidence of arrhythmia in the norepinephrine group was observed to be significantly lower than that in the control group (RR = 0.54; 95% CI, 0.45 ~ 0.64; *P* < 0.0001; Fig. 4a).

#### Effect of norepinephrine on the rate of achieving target MAP in patients

The rate of achieving target MAP was analyzed in 3 RCTs using a random-effect model (${I}^{2}$ = 92%) indicated no significant difference in the rate between the norepinephrine group and the control group (RR = 1.27; 95% CI, 0.72 ~ 2.26; *P* = 0.41; Fig. 4b).

## Discussion

In this study, 14 RCTs, involving 5028 cases, on the efficacy and safety of norepinephrine were included for meta-analysis. Corresponding results showed that the use of norepinephrine in patients with septic shock was negatively correlated with the 28-day mortality, length of stay in ICU, and incidence of arrhythmia. It suggests that norepinephrine can effectively reduce the mortality rate of patients with septic shock and alleviate their disease burden, accompanied by a relatively low incidence of side effects such as arrhythmia. However, this study failed to find any statistically significant differences in the improvement of in-hospital mortality rate, 90-day mortality rate, length of hospital stay, and rate of achieving target MAP between norepinephrine and other vasopressor agents in the treatment of septic shock.

By using the Cochrane risk of bias tool, the quality of evidence in outcome measures varied from moderate to high, but the risk of bias in each field of most trials was low or unclear. Only four trials showed a high risk of performance bias due to the open-labeled design in two studies [14, 25], and no blinding of the outcome measures in the remaining two studies [11, 12]. Except for one study with randomization based on odd or even days, all the other trials were classified as low-risk in terms of random sequence generation [25].

Our meta-analysis of in-hospital mortality rate, 90-day mortality rate, and length of hospital stay showed no significant difference between norepinephrine and other vasopressor agents. It was consistent with the previous two meta-analyses, which showed that norepinephrine did not have a significant advantage in controlling patient mortality compared to other vasopressor agents such as dopamine, adrenaline, and phenylephrine [9, 26]. However, meta-analysis based on studies of 28-day mortality rates revealed that norepinephrine can better control the mortality rate of septic shock patients compared to other vasopressor agents. It was similar to the conclusion reported in two reviews [27, 28], where norepinephrine was superior to dopamine in a 28-day mortality rate. It is speculated that compared with other vasopressor agents, norepinephrine can better control short-term mortality and timely alleviate the condition of illness, but requiring special attention to in-hospital care to lengthen the long-term life of patients.

There is still some controversy about the view that norepinephrine shortens hospitalization time and ICU hospitalization time [13, 14]. Septic shock is prone to severe circulatory, cellular, and metabolic abnormalities and has a high mortality rate [1]. Usually, patients with septic shock in the general hospital will be immediately transferred to the ICU for treatment. After the treatment is improved, they will be transferred out of the ICU ward. The time of treatment in the ICU is longer, but the time of hospitalization in the general ward is relatively short. During septic shock, norepinephrine should be given as early as possible, which is the key to the treatment of septic shock [6]. Therefore, norepinephrine may shorten the length of ICU stay, but the difference in length of hospital stay may not be obvious. Contrary to the results of the length of hospital stay, users of norepinephrine had a shorter length of stay in ICU compared to other vasopressin users, with a statistical difference. However, prior meta-analysis revealed the opposite results as well, revealing that there was no significant difference in the length of stay in the ICU among patients [20]. It may be explained by the inclusion of different studies, with more updated literature included in our meta-analysis than that in previous research. Meanwhile, our study also indicated that the use of norepinephrine resulted in better outcomes adverse reactions, with a lower incidence of arrhythmia in patients with norepinephrine.

Accumulated data supports that maintaining target MAP and initiating early use of vasoactive agents are associated with the reduction of mortality in patients with septic shock [29, 30]. The SSC Guidelines emphasize that hemodynamic stability and sufficient perfusion of major organs should be achieved within 1 h [31]. However, our meta-analysis showed that compared to other vasopressor agents, norepinephrine had no advantage in achieving target MAP. Moreover, there was significant heterogeneity (92%) in the results of achieving target MAP, which may be attributed to differences in the definition of achieving target MAP in different studies, the use of control drugs, and the dosage of drugs used. Due to the presence of significant heterogeneity, there might be no significant effect of norepinephrine on achieving target MAP. In addition, studies have found that in cases of refractory septic shock, other vasopressor agents can be applied to increase MAP in patients using norepinephrine [32, 33]. Similarly, in a prospective open-labeled study, patients who used other vasopressin and norepinephrine simultaneously achieved MAP of > 65 mmHg faster than patients receiving norepinephrine alone [34].

Patients with septic shock have a critical condition, and treatment and nursing measures need to be closely linked. Therefore, ICU nurses should be proficient in understanding the nursing risks of patients with septic shock, enhance risk prevention awareness, and promptly administer norepinephrine [8]. Meanwhile, during the use of norepinephrine, the use of a micro-injection pump can ensure a constant pump speed and maintain the steady-state blood drug concentration of norepinephrine. After administering vasopressor medication, dynamically evaluate the patient’s blood pressure and heart rate. When the mean arterial pressure is less than 65 mmHg, report to the doctor in a timely manner to determine the reason for increasing the infusion rate of vasopressor medication [7]. Early and effective fluid resuscitation in patients with septic shock is the key to reversing the condition. Effectively improving tissue perfusion, increasing oxygen delivery, and reducing organ failure, guided resuscitation based on hemodynamic evaluation can effectively improve prognosis [6]. As the executor of fluid resuscitation treatment, ICU nurses play an important role in determining whether patients need to continue fluid replacement. They can first choose indicators that quickly reflect hemodynamic conditions such as mean arterial pressure, heart rate, urine volume, and lactate value to determine whether fluid replacement is necessary [7]. In addition, continuous use of vasoconstrictors is an independent risk factor for secondary deep vein thrombosis (DVT) in the ICU; therefore, a combination of medication and mechanical prophylaxis should be used.

However, this study still has some limitations. Firstly, children were not included due to limited available trials. Secondly, with literature retrieval in three databases, only 14 trials met the inclusion criteria, which might affect the stability of the results. These findings shall be confirmed by more clinical studies in the future. Thirdly, there may be unpublished or inaccessible reports that were not included, although we believed that all relevant studies should be included. Collectively, the findings of this study still need further supplementary validation based on more high-quality RCTs involving different age groups.

## Conclusion

In conclusion, there is still not enough evidence to support that norepinephrine is superior to other vasopressor agents in terms of long-term mortality rate, achieving target MAP, and total length of hospital stay. However, the present meta-analysis highlights the superiority of norepinephrine in improving the 28-day mortality rate, shortening the length of stay in ICU, and reducing the incidence of arrhythmia, making it an effective and the safest option for septic shock. ICU nurses should be proficient in understanding the nursing risks of patients with septic shock, improve risk prevention awareness, and promptly administer norepinephrine. Anyway, larger-scale RCTs should be conducted to demonstrate the efficacy and safety of norepinephrine compared to other vasopressor agents. Additionally, it is recommended to perform appropriate allocation concealment and blinding to subjects and staff in future trials to reduce the risk of bias.

## Data Availability

All data generated or analyzed during this study are included in this published article.
